# Removal of the Liver!

**Published:** 1848-01

**Authors:** 


*Removal of the Liver!*—The eredulity of the public, and the incredulity of the Profession, were lately much excited by an announcement that a Dr. Thompson, residing near Columbus, Ohio, had extirpated a hypertrophied liver weighing some twenty or thirty pounds!! The victim was represented to be doing very comfortably without a liver! At length, however, the patient died, having lived sufficiently long to demonstrate the extraordinary fact that the hepatic viscus may be succesfully removed! A correspondent of the New-York Annalist, writing from the neighborhood of the place where this astonishing chirurgical feat was performed, furnishes an explanation of the affair. A large tumor, it appears, was removed from the abdomen, the operator, supposing, or, at all events, asserting that the tumor consisted of the liver, *and nothing else*, and that no vestige of the organ remained after the operation. After the decease of the patient, this redoubtable hero of the scalpel presided at an autopsy, having invited some spectators to testify to the verity of his achievement; when lo! the liver was found snugly and safely reposing in its usual locality, and the substance removed turned out to be an ovarian tumor! It would be libel upon an ancient and respectable fraternity, the primitive surgeons, to stigmatize this as an instance of *barber surgery*—it should be called simply *butchery*, without prefixing the adjective chirurgical.

